# A Novel Inhibitor IDPP Interferes with Entry and Egress of HCV by Targeting Glycoprotein E1 in a Genotype-Specific Manner

**DOI:** 10.1038/srep44676

**Published:** 2017-03-23

**Authors:** Myungeun Lee, Jaewon Yang, Eunji Jo, Ji-Young Lee, Hee-Young Kim, Ralf Bartenschlager, Eui-Cheol Shin, Yong-Soo Bae, Marc P. Windisch

**Affiliations:** 1 Department of Applied Molecular Virology, Hepatitis Research Laboratory, Institut Pasteur Korea, 696, Seongnam-si, Gyeonggi-do, 463-400, Republic of Korea; 2 Department of Biological Science, Sungkyunkwan University, Seobu-ro, Jangan-gu, Suwon-si, Gyeonggi-do, Republic of Korea; 3 Department of Infectious Diseases, Molecular Virology, University Hospital Heidelberg, Heidelberg, Germany; 4 German Center for Infection Research, Heidelberg University, Heidelberg, Germany; 5 Laboratory of Immunology and Infectious Diseases, Graduate School of Medical Science and engineering, KAIST, Daejeon, Republic of Korea

## Abstract

Despite recent advances in curing chronic hepatitis C (CHC), the high economic burden to therapy, viral drug resistance, difficult to treat hepatitis C virus (HCV) genotypes and patient groups are still of concern. To address this unmet medical needs, we devised strategies to identify novel viral interventions through target-free high-throughput screening of small molecules utilizing a phenotypic-based HCV infection assay. Thereby, a very potent (EC_50_ 46 ± 26 pM) iminodipyridinopyrimidine (IDPP) drug candidate was selected, and confirmed in primary human hepatocytes (EC_50_ 0.5 nM). IDPP mainly targets a post-attachment step of HCV without affecting endosomal acidification, prevents the secretion of infectious particles and viral cell-to-cell spread. The putative molecular target of IDPP is glycoprotein E1, as revealed by selection for viral drug resistance (Gly-257-Arg). IDPP was synergistic in combination with FDA-approved HCV drugs and inhibited pre-existing resistant HCV strains induced by today’s therapies. Interestingly, IDPP exclusively inhibited HCV genotype 2. However, we identified the genotype-specificity determining region in E1 and generated HCV genotype 1 susceptible to IDPP by changing one amino acid in E1 (Gln-257-Gly). Together, our results indicate an opportunity to provide an alternative treatment option for CHC and will shed light on the poorly understood function of HCV glycoprotein E1.

Since its discovery in 1989^1^, hepatitis C virus (HCV) has been recognized as a major global health problem that causes chronic liver diseases including cirrhosis and hepatocellular carcinoma (HCC)^2^. In recent years, direct-acting antivirals (DAAs) have been used to treat chronic hepatitis C^3^,^4^; however, there are still unmet medical needs imposed by high treatment costs, a large proportion of undiagnosed infections, and the emergence of drug resistance, although the latter is a rather rare event^4^,^5^,^6^. According to WHO guidelines (hepatitis C treatment guidelines, April, 2016), preferred regimens should be considered based on the genotype of HCV, and must be used at least one other DAA in a combinatorial therapy. Recently, a new drug application was submitted to the U.S. Food and Drug Administration (FDA) for the approval of a Sofosbuvir/Velpatasvir/Voxilaprevir combinatorial therapy (http://hepatitiscnewdrugresearch.com/). This therapy will be the first once-daily single tablet regimen available for patients infected with HCV genotypes 1–6.

However, further efforts are required to identify additional anti-HCV drugs with novel modes of action (MOA) in order to add value to existing therapies of chronic hepatitis C, e.g. by preventing reinfection of liver grafts, inhibiting the vertical transmission of the virus from mother to child or inhibiting drug resistance virus generated by first line of therapy.

HCV is a small (55–65 nm), enveloped, positive-sense single-stranded RNA virus belonging to the *Flaviviridae* family. The RNA genome encodes a single precursor polyprotein which is processed by host and viral proteases into 10 viral proteins^7^. The first generation HCV DAAs approved by the FDA target the viral NS3 protease (telaprevir and boceprevir) were already withdrawn from the market due to severe side effects^8^, whereas second-generation DAAs targeting NS5A phosphoprotein (daclatasvir)^6^, or the viral RNA-dependent RNA polymerase NS5B (sofosbuvir) have been approved recently^5^.

The discovery of the infectious HCV cell culture (HCVcc) system^9^,^10^ has enabled researchers to monitor the entire viral life cycle. This allows the discovery of drugs that target, in addition to RNA replication, early and late steps such as viral attachment and entry as well as assembly and egress of HCV particles^11^,^12^. Using the HCVcc system, small molecules, including ferroquine^13^, HCV II-1^14^, curcumin^15^ and green-tea polyphenolepigallocatechin-3-gallate (EGCG)^16^, have been identified as HCV entry inhibitors. Those molecules have been shown to target the viral glycoproteins E1 and E2. The E1-E2 heterodimer, eventually in a trimeric form^17^, is required to bind the entry factors CD81, SR-BI, Claudin-1, and Occludin^18^. Targeting viral entry offers the advantage of tackling a viral infection at its initial steps even before the virus starts to multiply its genomic material which also may reduce the emergence of viral drug resistance. Additionally, entry inhibitors are predestinated of preventing reinfection of liver grafts or inhibiting the vertical transmission of the virus. Accordingly, HCV envelope glycoproteins are desirable drug targets.

Here we describe an imaging-based strategy for a phenotypic high-throughput screening (HTS) assay using HCVcc expressing a NS5A-GFP fusion protein^19^. Identified hits were prioritized to affect only HCV entry as determined with the HCV pseudoparticle (HCVpp) system. Using this approach, we identified iminodipyridinopyrimidine (IDPP), and investigated the druggability of IDPP and identified a dual MOA that is associated with HCV glycoprotein E1.

## Results

### IDPP Inhibits HCV Infection

**Figure 1:**
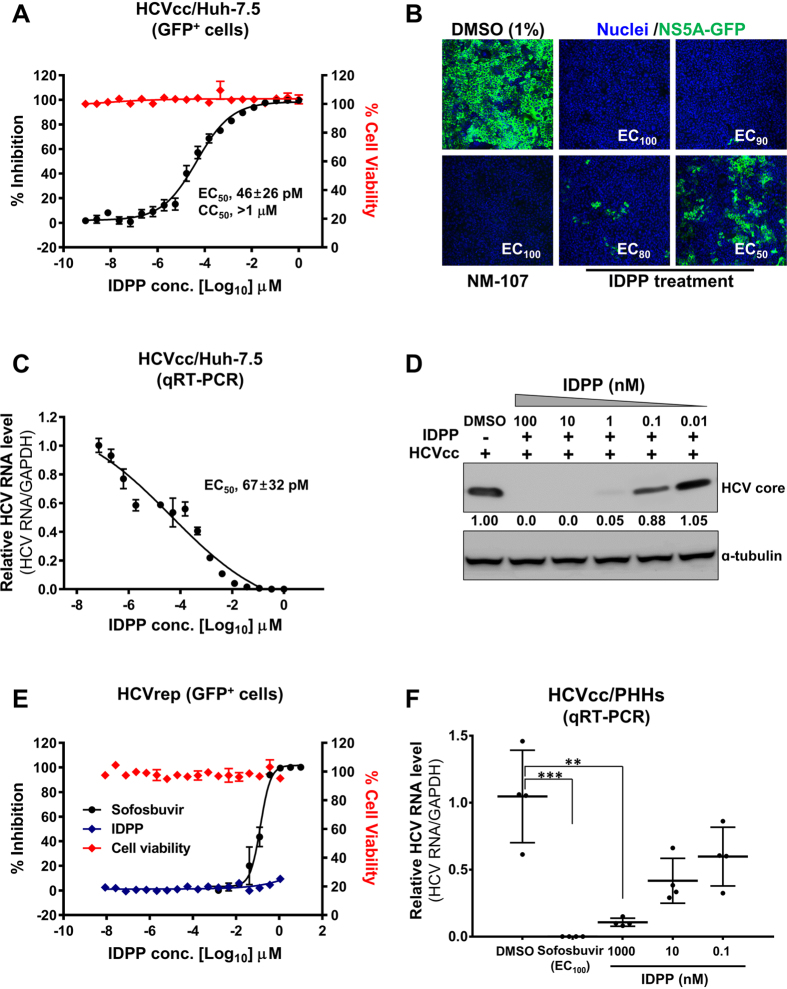
Anti-HCV activity of IDPP. (**A**) Anti-HCV activity in Huh-7.5 cells. Cells were pretreated with increasing concentrations of IDPP for 2 h followed by infection with HCVcc (JFH1) for 72 h in the presence of IDPP. Black squares and red squares indicate inhibition of HCV infection, and cell viability, respectively. The EC50 was determined by dose–response curve (DRC) analysis. Values represent the mean ± SD of five independent experiments in duplicate. (**B**) HCV replication and total cell number were assessed by determining the number of GFP-positive cells and blue nuclei for 3 days in the presence of inhibitors (IDPP and NM-107), respectively. Images were acquired by confocal microscopy at 200X. (**C**,**D**) DRC analysis was conducted as described above. (**C**) Intracellular HCV RNA levels were quantified by qRT-PCR and normalized to GAPDH mRNA (**D**) HCV core protein levels in lysates of infected cells were determined by Western blot analysis. (**E**) HCV subgenomic replicon cells were treated with IDPP and sofosbuvir. HCV replication was assessed by determining the number of NS5A-GFP-positive cells. (**F**) Evaluation of antiviral activity in PHHs. The inhibition of HCV infection was assessed by intracellular HCV RNA amounts quantified by qRT-PCR and normalized to GAPDH mRNA, ***p < 0.001 or **p < 0.01.

We screened more than 240,000 small molecules with the infectious HCVcc system^19^ and discovered IDPP (S1 Fig) as a potential anti-HCV drug candidate. The anti-HCV activity of IDPP was evaluated by a dose-response assay in the HCVcc system and fitted to a sigmoidal curve between the positive control 2′-C-methylcytidine (EC_100_ 100 μM) and the negative control (1% DMSO, no inhibition). In this way, we determined the EC_50_ of IDPP to be 46 ± 26 pM (Fig. 1A) and the cytotoxicity (CC_50_) to be 30 μM (S2 Fig). The expression of the NS5A-GFP fusion protein, which served as a marker for productive HCV infection, was inhibited by IDPP in a dose-dependent manner (Fig. 1B). We further confirmed the antiviral activity of IDPP by measuring intracellular HCV RNA levels by qRT-PCR (EC_50_ 67 ± 32 pM) (Fig. 1C) and by determining viral core protein expression (Fig. 1D). IDPP significantly reduced HCV RNA and core protein expression. However, RNA replication was not affected by IDPP as determined with a subgenomic replicon, while it was significantly inhibited by sofosbuvir (Fig. 1E), suggesting that IDPP interferes with HCV entry and/or viral production rather than genome replication. In addition, the anti-HCV activity of IDPP was confirmed in primary human hepatocytes (PHHs) with an EC_50_ value of approx. 0.5 nM (Fig. 1F).

### IDPP Inhibits HCV Entry

**Figure 2:**
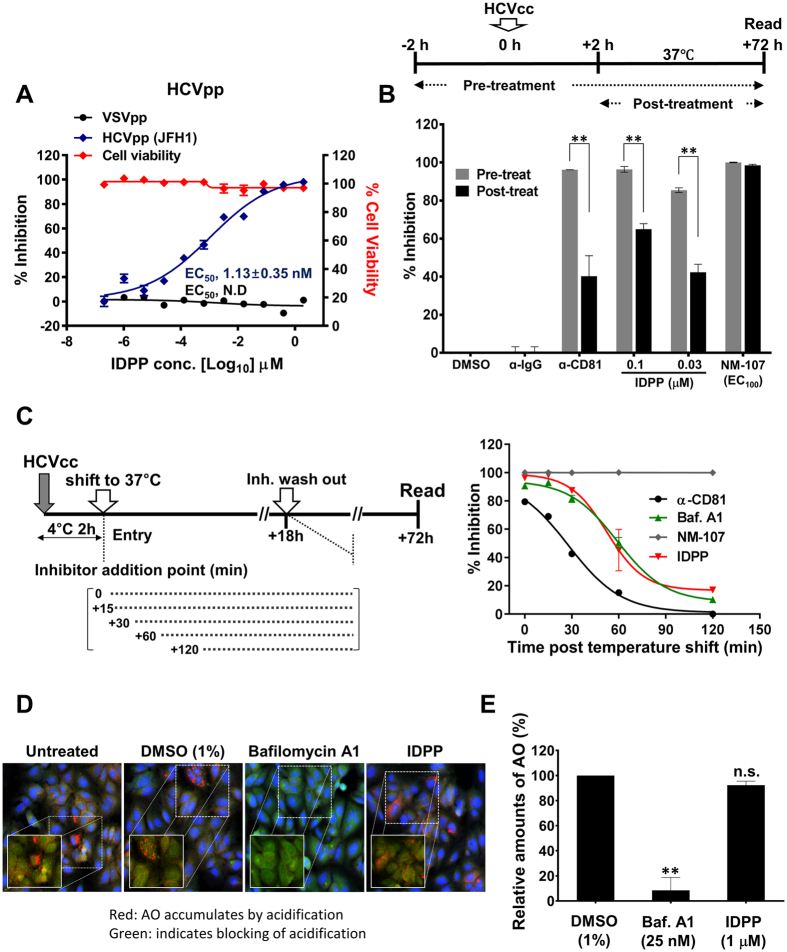
Inhibitory effects of IDPP on HCV entry. (**A**) Evaluation of IDPPs antiviral activity using HCV pseudoparticles (HCVpps). Transduction rates and percentage of inhibition were determined with HCVpp (JFH1) and VSVpp in the presence of IDPP. The means ± SD of duplicate experiments are shown. N.D = not determined. (**B**) Huh-7.5 cells were treated for 2 h with inhibitors before HCVcc infection (gray bars), or were treated for 2 h post-infection (black bars). At 72 h p.i., percent inhibition was calculated. Data represent means ± SD. **p < 0.01. (**C**) A Time-of-addition assay was performed as illustrated (detailed description in supplement). Data represent the mean ± SD of two independent assays in duplicate. (**D**) Evaluation of endosomal acidification by acridine orange staining. Images were acquired by confocal microscopy at 200X. (**E**) Quantification of endosomal acidification. Data are presented as the mean ± SD of two independent experiments. **p < 0.01; not significant (n.s.).

We next utilized HCV pseudoparticles (HCVpps) containing JFH1 glycoprotein E1/E2 heterodimers in the envelope and determined the effect of IDPP on HCV entry. IDPP treatment specifically inhibited HCVpp transduction (EC_50_ 1.13 ± 0.35 nM) (Fig. 2A), supporting the assumption that IDPP interferes with HCV entry. To investigate entry inhibition by IDPP in further detail, we treated cells with IDPP 2 h pre- and post-infection with HCVcc. Thereby, we observed that the inhibitory capacity of IDPP was significantly reduced when treatment occurred 2 h post-infection (*p* < 0.01), similar to α-CD81 mAb treatment, while no differences between pre-treatment and post-treatment with 2′-C-methylcytidine (NM-107, 100 μM) were observed (Fig. 2B). These data corroborate the observations made with HCVpps and replicons indicating that IDPP interferes with a step prior to HCV translation and replication.

To more precisely understand the entry inhibition, we performed time-of-addition experiments with IDPP as described in Supplementary Information. As shown in Fig. 2C, IDPP still showed 100% inhibition after binding of HCV particle to the target cells, suggesting that IDPP-mediated inhibition does not affect viral attachment to host receptor. In addition, the IDPP response curve was quite similar to that of bafilomycin A1, but was different from that of the α-CD81 antibody (Fig. 2C). These results also support that IDPP inhibits HCV by interfering with a post-attachment step without affecting viral attachment to its receptor. Bafilomycin A1 inhibits the fusion of the HCV envelope with the endosomal membrane by blocking endosomal acidification after attachment and endocytosis^14^,^20^,^21^. However, in an acridine orange (AO) staining assay^22^ we found that IDPP treatment did not interfere with AO-associated red fluorescence in the endosomal compartment of infected cells, while blocking acidification with bafilomycin A1 treatment was accompanied by a color change to green fluorescence (Fig. 2D), and the difference was statistically significant (Fig. 2E). This observation suggested that IDPP-mediated HCV entry inhibition is most likely not associated with blocking of endosomal acidification.

### IDPP Inhibits HCV Cell-to-Cell Spread

**Figure 3:**
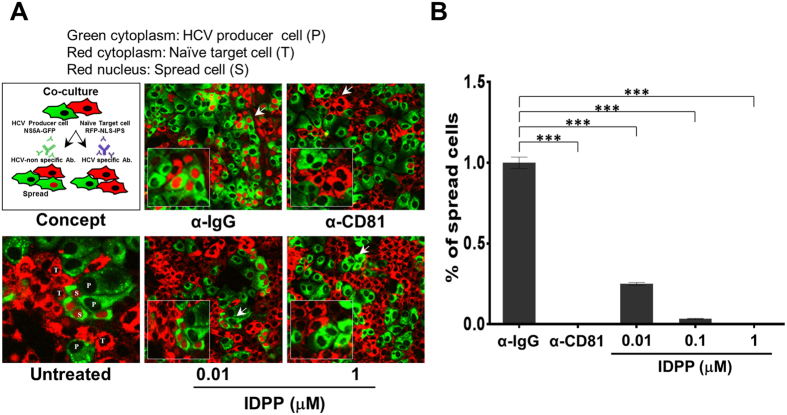
IDPP interferes with HCV cell-to-cell spread. (**A**) Viral spread assay was performed as described in concept image. After JFH1 (GFP-tagged) infected Huh-7.5 cells (producer cells, P) were co-cultured with naïve TagRFP-NLS-IPS cells (target cells, T), cell-to-cell spread of virus was assessed by counting of red nuclei (spread cells, S) (upper panel). P cells were co-cultured with T cells in the presence of IDPP or α-CD81 mAb (1 μg/mL), and S cells were assessed at 72 h post treatment. (**B**) S cells were counted and data are shown as the mean ± SD of four independent experiments. ***p < 0.001.

Generally, HCV can be transmitted not only by extracellular virus but also by intracellular infectious particles in a process termed cell-to-cell transmission. Cell-to-cell transmission is a key mechanism of persistent HCV infection^23^. We examined whether IDPP inhibited the cell-to-cell transmission of HCV using the visualized spread assay as described previously^24^. In this assay, we observed that IDPP was effective in inhibiting HCV cell-to-cell spread (Fig. 3A) and that the inhibitory effect was statistically significant (*p* < 0.001) (Fig. 3B). These results indicate that IDPP has the capacity to inhibit cell-to-cell transmission of HCV as well as cell-free viral infection.

### IDPP Interferes with the Secretion of Infectious HCV Particles

**Figure 4:**
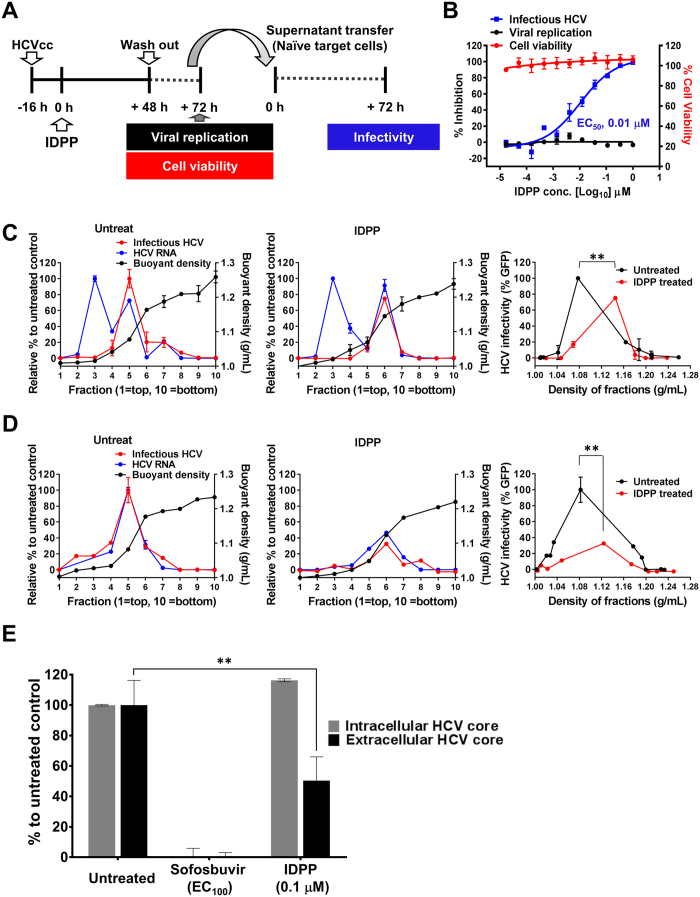
Inhibitory effect of IDPP on secretion of infectious HCVcc. (**A**) Schematic representation of the viral supernatant transfer experiment. Viral cell culture supernatants were transferred to naïve target cells and analyzed at 3 days post-infection. (**B**) Black circles, blue squares, and red circles indicate inhibition of viral replication, secretion of infectious HCV, and cell viability, respectively. Results are shown as the mean ± SD of two independent experiments in quadruplicate. Sucrose density gradient ultracentrifugation of cell lysates (**C**) and cell culture supernatants (**D**) obtained from transiently transfected HCV producer cells treated with IDPP or untreated (as described in supplement). Data represent relative HCV infectivity to DMSO (untreated control), HCV RNA levels, and buoyant density of each fraction. The mean value of two independent experiments is plotted and statistical significance of changes in HCV particle density calculated (**p < 0.01). (**E**) Total amounts of intra/extracellular HCV core in the transiently transfected cells were assessed by HCV core ELISA. Experiments conducted in duplicate and normalized to the untreated control. **p < 0.01.

When analyzing the HCVpp experiments, the EC_50_ value was significantly higher than what we observed in the HCVcc system (Fig. 2A). Additionally, the observation that IDPP retained an inhibitory effect stronger than a α-CD81 antibody when given 2 h post-infection (Fig. 2B and C) indicated that IDPP may act on additional steps in the HCV life cycle. Therefore, we examined the inhibitory effects of IDPP on the production of infectious virions by conducting cell culture supernatant transfer experiments (Fig. 4A) using transiently transfected cells, as previously reported^21^. IDPP treatment inhibited the production of infectious HCV particles in a dose-dependent manner without affecting HCV RNA replication. However, the inhibitory activity (EC_50_ 0.01 μM) was approximately 200-fold lower than what was observed in the HCVcc system (EC_50_ 46 pM) (Fig. 4B). To determine the impact of IDPP on the biophysical properties of intra- and extracellular infectious HCV particles and viral RNA, virions isolated from IDPP-treated HCV producer cells were analyzed by sucrose density gradient centrifugation. The levels of intracellular infectious viral particles and viral RNA were not affected by IDPP treatment (Fig. 4C). In contrast, the levels of extracellular infectious viral particles and viral RNA released from HCV producer cells were reduced by over 60% by IDPP treatment (Fig. 4D). However, the reduction of infectivity was not due to the carry-over of residual IDPP in the second infection assay, which was demonstrated by cell-free incubation of IDPP and HCVcc followed by ultracentrifugation. Thereby, we did not observe any virucidal effects or compound carry-over (S3 Fig). In addition, the amount of extracellular core protein was also significantly reduced by IDPP treatment, while the intracellular core protein level was slightly increased by IDPP treatment (Fig. 4E). These data suggest that IDPP also targets the later stages of the HCV life cycle, such as assembly, maturation, and/or release steps, without inhibiting viral genome replication and viral protein expression, resulting in the reduction of infectivity seen in Fig. 4D.

We noted that the buoyant densities of the fractions containing both intracellular and extracellular viral particles were increased from 1.08 g/mL to 1.14 g/mL in IDPP-treated cells (Fig. 4C and D). Similar patterns of HCV buoyant density changes were reported in apolipoprotein E (ApoE)-knockdown cells^25^. ApoE plays a critical role in the production of infectious HCV particles during the assembly and maturation steps^25^,^26^,^27^. We found intracellular ApoE accumulation even in HCV-infected cells when cultures were treated with IDPP (S4 Fig). Our preliminary data led us to propose that intracellular accumulation of ApoE following IDPP treatment might be accompanied by reduced release of HCV virions and a shift of viral particle towards higher density.

### IDPP Targets the Envelope Glycoprotein E1 in a Genotype-Dependent Manner

**Figure 5:**
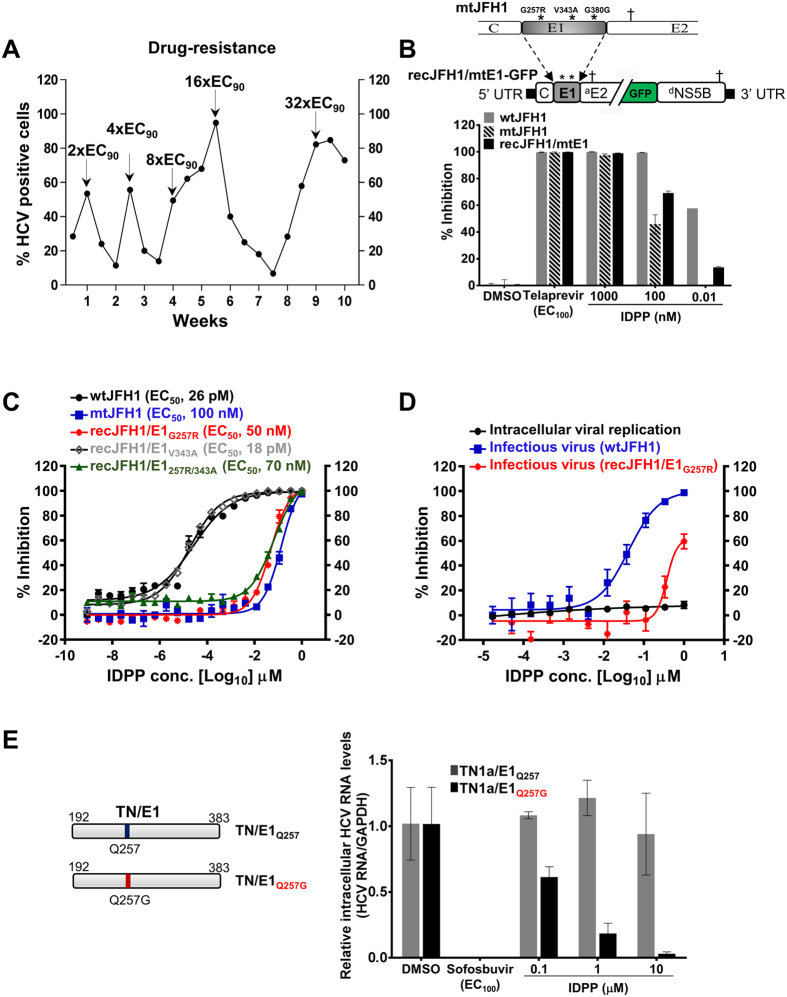
Viral IDPP resistance study and generation of IDPP-susceptible HCV genotype 1 variant. (**A**) IDPP-resistant HCV was created by culturing of JFH1-GFP virus-infected Huh-7.5 cells with increasing compound concentrations (2-fold) as indicated. (**B**) Three mutations in glycoprotein E1 were identified and are indicted by asterisks ‘*’ (upper). Resistance to IDPP was evaluated with recombinant viruses (lower). (**C**) DRC analysis with IDPP-recombinant viruses including the given amino acid substitutions. (**D**) Supernatant transfer assay with parental (wtJFH1) and IDPP-recombinant virus (recJFH1/E1G257R). (**E**) Generation of IDPP-susceptible genotype 1 virus (TN) by amino acid substitution (Q257G) in HCV E1 glycoprotein. At 72 h p.i., intracellular HCV RNA levels were determined by qRT-PCR. Relative HCV RNA levels normalized to GAPDH are shown.

To identify the putative molecular target of IDPP, we generated drug-resistant HCV by culturing infected cells in the presence of stepwise increasing concentrations of IDPP. The concentration was increased from 0.01 μM (2x EC_90_) to 0.32 μM (32x EC_90_) over a period of 10 weeks (Fig. 5A). Through serial cultures of infected cells, we isolated IDPP-resistant HCV and subjected the viral RNA to whole-genome sequencing. IDPP-resistant virus (mtJFH1) was found to have two non-synonymous (coding-change) mutations at positions 257 (G257R) and 343 (V343A) and one synonymous mutation at 380 (G380G) in glycoprotein E1 (Fig. 5B, upper). When the mutant E1 gene (mtE1) was introduced into the parental genome, the recombinant virus (recJFH1/mtE1) was also resistant to IDPP treatment (Fig. 5B, lower). In order to more precisely identify the IDPP target site, single or double coding-change mutations were introduced into the parental genome by site-directed mutagenesis (recJFH1/E1_G257R_, recJFH1/E1_V343A_, and recJFH1/E1_257R/343A_). To rule out differences in viral fitness of IDPP-recombinant virus, we compared its relative infectivity to the parental construct and did not observe significant differences (S1 Table). Subsequently, IDPP resistance was confirmed by DRC analysis, which demonstrated that the G257R mutation in E1 rendered the JFH1 strain resistant to IDPP and shifted the EC_50_ value by more than 1,000-fold (EC_50_ 0.05–0.1 μM), while the V343A mutation had no impact on drug resistance (EC_50_ 18 pM) (Fig. 5C). Furthermore, we confirmed that the IDPP-resistant mutation G257R affected the secretion of infectious HCV particles from full genome-transfected cells by observing an approximately 10-fold shift in the EC_50_ value (0.04 to 0.37 μM) (Fig. 5D). In order to elucidate the importance of glycoprotein E1 position 257 for IDPP antiviral activity, we replaced the glycine residue (Gly-257) with alanine (G257A), aspartic acid (G257D), or serine (G257S) and compared the mutants to the wild type virus and the IDPP-resistant mutant (G257R) (S5 Fig). Upon transfection, we measured HCV RNA replication and the production of infectious HCV particles. None of the substitutions had an impact on viral RNA replication (S5B Fig), and the amount of viral RNA released into the culture supernatant remained unchanged or even increased in the mutants, indicating efficient virus production (S5C Fig). However, inoculation of naïve Huh-7 target cells clearly demonstrated that amino acid substitutions G257A, G257D, and G257S significantly reduced or completely abolished infectivity (S5D Fig). In addition, the G257D mutation conferred IDPP resistance, albeit to a lower extent than did the G257R substitution (S5E Fig).

Because G257 is located in a non-conserved E1 region, we wondered if other HCV genotypes and subtypes can be inhibited by IDPP. Therefore, a panel of HCV chimeras expressing the structural proteins of seven different genotypes (gt) and subtypes^28^ were evaluated. IDPP very efficiently inhibited JFH1 (EC_50_ 60 pM), however the antiviral effects on Jc1, a J6(gt2a)/JFH1(gt2a) chimera and on J8, a J8(gt2b)/JFH1(gt2a) chimera, were reduced by 67-fold and 833-fold, respectively (Table 1). Furthermore, other HCV genotypes (Table 1) and dengue viruses (DENV) were not inhibited by IDPP (S6 Fig), demonstrating that the effect of IDPP is specific to HCV gt2. However, we wondered whether G257 introduced into E1 glycoprotein of genotype 1 could lead to susceptibility to IDPP. Accordingly, we performed site-directed mutagenesis in E1 of the gt1 TN isolate (Q257G). Remarkably, this recombinant gt1a (TN/E1_Q257G_) virus became sensitive to IDPP in a dose-dependent manner (EC_50_ < 0.5 μM) (Fig. 5E). These data clearly suggest that IDPP targets G257 in the E1 glycoprotein of HCV gt2.

### Synergistic Antiviral Effect of IDPP in Combination with Selected DAAs or IFN-α

To evaluate the possibility of using IDPP as a new drug class suitable for combination with already existing DAAs or IFN-α, we determined the antiviral activity of IDPP in combination with telaprevir, daclatasvir, sofosbuvir, or IFN-α. Drug combinations were analyzed by dose-response matrices and the combination index (CI) of each individual IDPP-drug combination was calculated according to the methods of Chou *et al*. and analyzed using CompuSyn software^29^. IDPP showed considerable synergism in combination with daclatasvir (CI = 0.50), synergism with telaprevir (CI = 0.87 ± 0.01), and was additive with sofosbuvir (CI = 1.00 ± 0.04) (Table 2). Notably, IDPP demonstrated the strongest synergistic effect in combination with IFN-α (CI = 0.26) (Table 2). These results suggest that IDPP is a suitable alternative drug for HCV gt2 infections and can be used in combination therapy with approved DAAs as well as IFN-α.

### Antiviral Effect of IDPP on DAA-Resistant HCV

Treatment of chronically HCV-infected patients with DAAs can select for drug resistant viral variants^30^,^31^. To demonstrate that IDPP is capable of inhibiting drug-resistant HCV, we evaluated several DAA- (telaprevir, daclatasvir, and sofosbuvir) resistant HCV mutants for sensitivity to IDPP. To this end, we used the NS3 mutant A156T (resistant to telaprevir and several other protease inhibitors), the NS5A mutant L31M (resistant to daclatasvir and ledipasvir), and the NS5B mutants T179A/S282T/M289L/I293L (resistant to sofosbuvir). Of note, all tested DAA-resistant viruses were fully sensitive to IDPP treatment (Table 3). On the other hand, the IDPP-resistant viruses shown in Fig. 5 were sensitive to DAAs in both infected and transfected cell culture conditions (S7 Fig). These results demonstrate that IDPP can efficiently control drug-resistant mutants generated by today’s HCV treatment regimen when used in combination with approved DAAs.

## Discussion

We devised strategies utilizing the infectious JFH1 cell culture system^10^,^11^,^12^, screened for small molecule inhibitors with novel MOAs, and identified IDPP as a potential drug candidate for chronic hepatitis C. IDPP showed very potent antiviral activity (EC_50_ 46 ± 26 pM) against HCV gt2 (Fig. 1) without causing cytotoxicity (CC_50_ 30 μM, S2 Fig). The outstanding potency of IDPP is likely due to targeting two independent steps in the HCV life cycle. Similar potencies have been observed with NS5A inhibitors inhibiting HCV RNA replication and RNA encapsidation^6^. Next, we scrutinized the MOA of IDPP by conducting HCVpp (Fig. 2A) and time-of-addition experiments (Fig. 2B and C), which demonstrated that the inhibitory effect of IDPP was associated with an ability to target the late entry step during the HCV life cycle, but was unlikely to affect endosomal acidification such as that occurring after treatment with bafilomycin A1 (Fig. 2D). Cell-to-cell transmission, the major route of HCV transmission in the liver, was also efficiently blocked by IDPP (Fig. 3)^32^,^33^. However, IDPP did not demonstrate any inhibitory effect on intracellular viral RNA replication or viral protein expression (Figs 1E, 4C and E), suggesting that the MOA of IDPP is different from that of the current FDA-approved HCV DAAs. In addition, IDPP treatment reduced the titer of extracellular infectious virions, HCV core amounts and v-RNA in the culture supernatants of transfected HCV producer cells, while the buoyant density of the viral particle was increased (Fig. 4D and E), suggesting that IDPP also interferes with late assembly and/or egress steps of the infectious virion. It is well established that ApoE plays a critical role in production of infectious HCV particles during the late assembly and maturation steps^26^,^27^,^34^,^35^, and similar HCV buoyant density changes were reported in ApoE-knockdown cells^25^. We found that IDPP treatment restored normal intracellular ApoE levels in HCV-infected cells (S4 Fig), suggesting that the intracellular retention of ApoE might be associated with the increased buoyant density and lower release of the extracellular viral particles. Alternatively, the density shift may simply be due to lower association of HCV with lipids, which could be mediated by E1 rather than ApoE.

Next we sought to identify the molecular target of IDPP by generating IDPP-resistant HCV mutants, and found that HCV envelope glycoprotein E1 was targeted, resulting in an EC_50_ value shift by more than 1,000-fold (Fig. 5A and B). Sequencing analysis and site-specific mutagenesis revealed that the G257R mutation in E1, which lies outside of the conserved fusion peptide-like motif that is essential for viral entry^36^, conferred resistance to IDPP (Fig. 5C and D). Even though IDPP exclusively inhibited HCV gt2, when the amino acid substitution Q257G was introduced into E1 of HCV gt1, the recombinant virus became susceptible to IDPP (Fig. 5E). Interestingly, none of the HCV genotypes except gt2a has Gly-257 in the E1 glycoprotein (Table 1), which likely explains why HCV inhibition by IDPP was restricted to gt2a, suggesting that the domain of E1 carrying the resistant mutation likely plays a crucial role in HCV gt2 entry (S5 Fig).

HCV envelope glycoproteins are crucial viral determinants which have not yet been sufficiently exploited as drug targets. Until to date, all marketed DAAs interfere with HCV RNA replication, however in order to prevent reinfection of transplanted liver or to prevent vertical virus transmission entry inhibitors are desirable. Previously, several entry inhibitors, mainly derivatives of natural products, have been reported^13^,^14^,^37^; however, most of these inhibitors target HCV glycoprotein E2^14^,^21^ rather than E1. Recently, a small molecule inhibitor, EI-1, which targets the E2 glycoprotein of exclusively HCV gt1 and blocks HCV entry and cell-to-cell transmission of virions, was described^21^. Furthermore, ferroquine was shown to interefere with HCV entry by targeting glycoprotein E1 (S327A), but did not interfere with egress of infectious HCV particles, and at higher concentrations also inhibited HCV RNA replication^13^. More recently, it was reported that flunarizine prevented membrane fusion of HCVcc in the late endosome, but was inactive on HCVpp and also did not interfere with viral egress. Flunarizine’s putative molecular target is glycoprotein E1 at position Q289, within the potential fusion peptide which is conserved among all HCV genotypes, but flunarizine preferentially inhibited HCVcc gt2^38^. The effects of combining these E1-targeting drugs with IDPP remain to be addressed.

The strong synergism of IDPP with all the tested HCV drugs not only supports its use in combination therapy and clearly indicates that IDPP inhibits HCV through a different mechanism, such as targeting viral entry and egress. Furthermore, inhibitors like IDPP provide a novel approach to treat and prevent chronic hepatitis C, e.g. preventing reinfection of liver grafts or inhibiting the vertical transmission of the virus from mother to child. Because of IDPPs high antiviral potency a shortened treatment period could reduce costs by maintaining high sustained virologic response (SVR). In order to evaluate drug-like properties of IDPP, the half-life of the compound was determined by a metabolic stability assay utilizing human liver microsomes. Thereby, the half-life was calculated to be 54.7 minutes which is metabolically stable, but not too stable which otherwise might lead to toxic drug accumulation (data not shown).

In conclusion, IDPP, a new picomolar inhibitor of HCV, is described that acts on viral entry and egress steps by targeting glycoprotein E1 of genotype 2. IDPP acts synergistically with HCV drugs and inhibits DAA-resistant HCV variants, suggesting that IDPP potentially could add value to existing therapies by reducing the emergence of drug-resistant mutants, inhibiting pre-existing resistant strains, and by preventing reinfection of liver grafts. IDPP is a potential HCV drug candidate, blocking a viral envelope protein without affecting viral attachment which is a novel paradigm in antiviral therapy. Further studies on the structure of E1 and the molecular interactions between IDPP and E1 will provide a better understanding of the MOA of IDPP and will shed light on the poorly understood function of HCV glycoprotein E1.

## Materials and Methods

### Cell lines and cell culture

Huh-7.5^39^ cells and HCV reporter cells expressing RFP-NLS-IPS^40^ were kindly provided by Charles M. Rice (Rockefeller University, New York, USA). The cell lines were cultured in Dulbecco’s modified eagle medium (DMEM) supplemented with 10% fetal bovine serum (FBS) (PAA), 10% nonessential amino acids (NEAA), 2 mM L-glutamine, 100 units/mL penicillin, and 100 units/mL streptomycin. Huh-7 derivative cell lines^41^ and Huh-7 cells harboring the HCV subgenomic replicons^9^ were kindly provided by Dr. Ralf Bartenschlager, (University of Heidelberg, Germany) and maintained in complete DMEM containing 0.6 mg/mL G418 (Geneticin, Gibco). Primary human hepatocytes (PHHs) were purchased from Invitrogen (Thermo, catalog number; HMSPIS). The cells were centrifuged at 100 xg for 10 min in hepatocyte thaw medium (Gibco, catalog number; CM7500) and plated in collagen coated 24-well plates (Thermo, catalog number; A11428-02). PHHs were maintained in Williams E Medium (Gibco, catalog number; A12176-01) containing cell maintenance supplement reagents (Gibco, catalog number; CM4000).

### Compounds and antibodies

IDPP was synthesized at Institut Pasteur Korea. Synthetic compound was dissolved in dimethyl sulfoxide (DMSO) at a concentration of 20 mM and further diluted in cell culture medium for the studies. NM-107 (2′-C-methylcytidine), VX-950 (telaprevir,), BMS-790052 (daclatasvir), and PSI-7977 (sofosbuvir) were purchased from Selleckchem, Inc. and Acme Bioscience, Inc. Bafilomycin A1 was purchased from Sigma (cat. B1793). Anti-CD81 (BD Biosciences, JS-81 clone cat. 555675), anti-HCV core (AB Chem cat. ab2740) and anti-ApoE (Santa Cruz biotechnology cat. sc-53570) monoclonal antibodies (mAbs) were purchased from the manufacturers.

### Plasmid construction

In order to generate the JFH1 E2p7-5A/5B-GFP construct, cell culture adaptive mutations were inserted into JFH1 E2 and p7 at positions N417S and N765D, respectively, as described previously^42^. The given amino acid positions refer to the JFH1 consensus genome (DDBJ/EMBL/GenBank accession no. AB047639)^43^. Emerald GFP was inserted into the JFH1 NS5A coding region at position 2360 as described previously^19^. This recombinant plasmid was used as a template for PCR-based site-directed mutagenesis to introduce further cell culture adaptive mutations with the following primer sets: Mut1_NS5A (V2153A) forward, 5′ GTC TCG TTC TGC GCT GGG CTT AAT TCC TAT G 3′ and Mut1_NS5A (V2153A) reverse, 5′ CAT AGG AAT TAA GCC CAG CGC AGA ACG AGA C 3′; Mut2_NS5A (V2440L) forward, 5′ GAG GAC GAT ACC ACC TTG TGC TGC TCC ATG 3′and Mut2_NS5A (V2440L) reverse, 5′ CAT GGA GCA GCA CAA GGT GGT ATC GTC CTC 3′; and Mut3_NS5B (V2941M) forward, 5′ GCG CCA CCC CTC AGG ATG TGG AAG AGT CGG 3′ and Mut3_NS5B (V2941M) reverse, 5′ CCG ACT CTT CCA CAT CCT GAG GGG TGG CGC 3′. All mutagenesis was performed using the Quikchange site-directed mutagenesis kit (Stratagene) according to the manufacturer’s instructions.

### Production of HCVcc particles

The full-length HCV RNA genome was synthesized by *in vitro* transcription using the RiboMAX Large Scale RNA Production System-T7 kit (Promega_P1300) according to the manufacturer’s protocol. Cell culture-derived infectious HCV (HCVcc) was produced by transfection of Huh-7 derivative cells as reported previously^41^ with HCV RNA transcripts of a cell culture-adapted JFH1 genome containing an NS5A-GFP fusion protein (JFH1_5 A/5B_GFP), an engineered JFH1 genome harboring viral titer-enhancing mutations in E2 and p7 (JFH1 E2p7_5 A/5B_GFP), or a site-directed mutated JFH1 genome with mutations in the E1 region (G257R, V343A, or G257R and V343A). In brief, 20 μg of each genomic HCV RNA transcript was mixed with Huh-7 derivative cells (6 × 10^6^) in a 0.4 cm gap cuvette, and the mixture was electroporated at 270 V and 975 μF using a Bio-Rad GenePulser II instrument. HCVcc particles expressing an NS5A-GFP fusion protein were harvested at 2, 3, and 4 days post-transfection. Viral supernatant was clarified by filtration using a syringe filter with a 0.2 μm pore size (Millipore, Bedford, MA) and stored at −80 °C as a viral stock. Determination of infectious viruses was done by tissue culture infectious dose 50 (TCID_50_) analyses as described previously^44^.

### Determination of antiviral activity

The antiviral activity of IDPP was assessed using cell culture-derived infectious HCV (HCVcc) expressing an NS5A-GFP fusion protein in the presence of inhibitors as previously reported^19^. Briefly, Huh-7.5 cells were seeded in 384-well plates (2.5 × 10^3^ cells/well) and inoculated with HCVcc. IDPP were serially diluted in complete DMEM (1 × 10^−9^ μM to 1 μM), added to each well of the plates, inoculated with HCVcc and incubated at 37 °C for 3 days. On day 3 post-infection (p.i.), cultured cells were fixed with 2% paraformaldehyde in PBS containing 10 μg/mL Hoechst 33342 (life technologies) for 30 min. HCV replication was analyzed by determining the number of GFP-positive cells using fully automated confocal microscopy (200X, Image press Ultra/Molecular Devices) or by measuring intracellular HCV RNA levels. HCV RNA was isolated with a CellAmp direct RNA prep kit (Takara cat.3732) and the level of HCV RNA was determined by quantitative real-time reverse-transcription polymerase chain reaction (qRT-PCR) using an RT-PCR kit (Takara cat.RR064A) with an HCV primer set (forward 5′-CGGGAGAGCCATAGTGG-3′ and reverse 5′-AGTACCACAAGGCCTTTCG-3′) and an HCV probe (5′-Texas Red-CTGCGGAACCGGTGAGTACAC-BHQ-2-3′). Cell viability was used as a marker for cytotoxicity by measuring the reduction in the cell population after drug treatment compared to untreated control cells. Percent inhibition was calculated using the values of maximum infectivity and background derived from infected cultures treated with 1% DMSO (0% inhibition) and with the EC_100_ of NM-107, an NS5B nucleoside polymerase inhibitor (100% inhibition) as references, using the formula [(DMSO - Sample) / (DMSO - EC_100_) x 100]. The antiviral activity of IDPP and other compounds was determined from the dose–response curve (DRC) and Hill equation analysis using Prism v. 5.0c software (Graph Pad Software, Inc., La Jolla, CA), and represented as 50% effective concentrations (EC_50_) and 50% cytotoxic concentrations (CC_50_).

### Western blot analysis

Western blot analysis was performed as described previously^44^ with some modifications. HCVcc infected cells were washed with Dulbecco’s phosphate buffered saline (DPBS) (Welgene. Inc., Korea) and lysed using radioimmunoprecipitation assay (RIPA) buffer containing 0.5% protease inhibitor cocktail (Sigma, St. Louis, MO). The cell lysate was clarified by centrifugation. Total protein content was determined by the Bradford assay (Bio-Rad). Equal amounts of protein were subjected to sodium dodecyl sulfate polyacrylamide gel electrophoresis (SDS-PAGE) and electro-transferred to a PVDF membrane. The membrane was blocked in Tris-buffered saline/T (0.1% Tween20) containing 5% skim milk for 2 h and then incubated at 4 °C overnight with primary antibodies (anti-HCV core or anti-ApoE). Membranes were washed and incubated with secondary antibody (Goat anti-IgG HRP) containing 1% skim milk for 1 h at room temperature. After washing, signals were detected with the enhanced chemiluminescence system LAS-3000 (FUJIFILM).

### HCV inhibition assay in primary human hepatocytes (PHHs)

The antiviral activity of IDPP was assessed by cell culture derived infectious HCV (HCVcc-JFH1) in PHHs. Briefly, PHHs cells were seeded in 24-well plates (3.5 × 10^5^cells/well) and inoculated with HCVcc (MOI = 5). Cells were pretreated for 2 h with three different concentrations of IDPP (1000 nM, 10 nM and 0.1 nM), the compounds were serially diluted in Williams E Medium andincubated at 37 °C for 2 days. On day 2 post infection (p.i.), HCV replication was analyzed by measuring intracellular HCV RNA level as described above. Percentage of inhibition was normalized with the values of maximum infectivity (1% DMSO) and 100% inhibition (EC_100_ of replication inhibitor; sofosbuvir) which were derived from controls.

### HCV entry inhibition and time-of-addition assays

To elucidate the effect of compounds on the early stage of HCV infection, Huh-7.5 cells in 384-well plates were pretreated with IDPP (0.1 μM or 0.03 μM), with anti-CD81 Ab (5 μg/mL), or with an NS5B nucleoside polymerase inhibitor (100 μM), followed by infection with HCVcc for 2 h. In order to further characterize early inhibition, a time-of-addition assay was performed following a previously reported protocol^20^ with minor modifications. In brief, GFP-expressing recombinant HCV (HCVcc-GFP) was inoculated with naïve Huh-7.5 cells at 4 °C for 2 h. Unbound virus was removed by washing with cold PBS, the infected cultures were shifted to 37 °C to allow for synchronous infection, and then IDPP or other inhibitors (10 nM bafilomycin A1 or a replication inhibitor, 100 μM NM-107) were added at 0, 15, 30, 60, and 120 min post-temperature shift. After 18 h of 37 °C incubation, the inhibitor-containing medium was replaced by fresh medium. The antiviral activity of each compound was quantified after 3 days by counting GFP-expressing cells. Data were normalized to controls as described above and statistical significance was set at *p* < 0.05.

### Acridine orange staining

Acridine orange staining was performed following a protocol described previously^22^. Briefly, Huh-7.5 cells were either left untreated or were treated for 72 h with IDPP (1 μM) or bafilomycin A1 (25 nM). Cells were then incubated for 20 min at 37 °C with complete medium supplemented with 5 μg/mL Acridine orange and analyzed immediately by confocal microscopy. Acidification was determined by quantifying intracellular red speckles using in-house image analysis algorithms IM 3.0 software at Institut Pasteur Korea (IPK).

### Inhibition assay with HCV pseudoparticles (HCVpps)

HCV E1E2-pseudotyped [or Vesicular stomatitis virus glycoprotein (VSVG) pseudotyped as a control] lentiviral particles expressing a *Firefly* luciferase (F-Luc) reporter gene were co-transfected using Lipofectamine 2000 as described previously^45^,^46^. In brief, 293 T cells seeded for 1 day in T-75 flasks were co-transfected with 5 μg of an HCVE1/E2 envelope protein expression vector (JFH1) or a VSV envelope expression vector^46^, 15 μg of a human immunodeficiency virus Gag-Pol expression packaging vector^46^, and 20 μg of a *Firefly* luciferase reporter transfer vector. The medium was replaced 6 h after transfection. Supernatants containing the pseudoparticles were harvested at 72 h and used as HCVpps in the entry inhibition assay as described above. Transduction rates and percentage of inhibition were determined by DRC analysis with HCVpp (JFH1) and VSVpp in the presence of IDPP (2 × 10^7^ μM to 2 μM).

### HCV replication assay

HCV subgenomic replicon cells^9^ (provided by Ralf Bartenschlager, University of Heidelberg, Germany) were used to examine the effect of compounds on the replication of HCV genomic RNA. Replicon cells seeded in 384-well plates were treated with compounds for 72 h. The inhibitory effect of the compounds on HCV RNA replication was monitored together with RNA polymerase inhibitor (sofosbuvir) as a reference compound.

### Sucrose density gradient centrifugation assay

For sucrose density gradient analysis, 20 to 80% sucrose density gradients were prepared with Optiprep Density Gradient (Sigma cat# D1556) and then each sample was loaded on top of a pre-formed 20 to 80% sucrose gradient according to a method described previously^25^. Gradients were ultracentrifuged at 120,000 xg for 16 h at 4 °C in an SW-41 Ti rotor (Beckman). Ten fractions (1 mL/fraction) from each gradient were collected from the top to the bottom of the tubes and the buoyant densities of the fractions were determined. HCV RNA from each fraction was extracted with TRIzol LS reagent (Invitrogen) and quantified by RT-qPCR as described above. The titer of infectious HCV in each fraction was determined by measuring GFP-positive cells after the 2^nd^ infection. In order to prepare the intracellular HCV particles, infected cells cultured in the presence of 1% DMSO, sofosbuvir (2 μM), or IDPP (0.1 μM) were washed with DPBS and trypsinized to remove cell surface HCV and then centrifuged for 5 min at 1100 rpm. Cell pellets (8 × 10^6^) were resuspended in 1 mL of DMEM containing 5% FBS and lysed by repetitive freeze/thaw cycles using liquid nitrogen and a 37 ^o^C water bath. Lysates were centrifuged at 12,000 rpm for 10 min at 4 °C to remove cell debris and then layered on top of a pre-formed 20 to 80% sucrose gradient. Likewise, to measure the amount of extracellular infectious HCV particles, the HCV culture supernatants derived from the same samples were collected and concentrated with Amicon (Millipore cat# UFC910096). All samples were ultracentrifuged as described above.

### Determination of intra/extracellular HCV core

Huh-7 derivative cells were transiently transfected with viral genome (JFH1). Two days later, transfected cells were seeded on T-75 flask. Next day, cells were treated with IDPP (0.1 μM) for 72 h. The amounts of intracellular and extracellular HCV core were determined by HCV core ELISA immunoassay. In order to prepare the intracellular core samples, cells were washed twice with PBS and lysed by addition of 0.2 mL PBS containing 1% Triton X-100 and protease inhibitor. Lysates were cleared by centrifugation at 11,000 rpm for 10 min at 4 °C. Likewise, to measure the amount of extracellular HCV core, viral particles in the culture supernatants of the same cultures were precipitated by the addition of PEG_8000_/NaCl solution to make final concentrations of 8% PEG_8000_ and 1.75 M NaCl, and stored at 4 °C overnight as reported^47^, followed by centrifugation at 2500 rpm for 45 min. HCV core protein was quantified using a commercial HCV core antigen detection ELISA assay kit (XpressBio Life Science Products, cat. No. XC-1000, USA) following the manufacturer’s instructions.

### Isolation of drug-resistant HCVcc *in vitro*

Huh-7.5 cells were infected with HCV gt2a (JFH1-GFP) virus in the presence of EC_90_ drug concentration. Either infected cells or supernatants were passaged with selective drug pressure. The process was repeated with stepwise (2-fold) increases in drug concentration until the breakthrough of resistance (32-fold EC_90_) as published before^48^,^49^. The replication of resistant virus in the presence of high-dose drug was monitored by counting the number of GFP-positive cells in each passage. Eventually, drug-resistant HCV was isolated. The viral RNA was extracted with TRIzol solution (Invitrogen, Carlsbad, CA) and amplified by RT-PCR with an HCV-specific forward primer (5′-TGCGAAAGGCCTTGTGGTACTG-3′) and reverse primer (5′-CCTCGTGTTTGCTGGGCATAAG-3′) using a QuantiTect Reverse Transcription Kit (Qiagen). The PCR products were then sequenced (Macrogen, Korea).

### *In vitro* site-directed mutagenesis

In order to introduce specific mutations (G257R and V343A) into the HCVcc E1 glycoprotein, we performed *in vitro* site-directed mutagenesis as reported previously^50^ with the following mutagenic primers: G257R mutation forward, 5′-CCCTCACGCAGCGTCTGCGGACG-3′ and reverse, 5′-CGTCCGCAGACGCTGCGTGA GGG-3′; and V343A mutation forward, 5′-CGCGTCCCCGAGGCCATCATAGACATCG-3′ and reverse, 5′-CGATGTCTATGAT GGCCTCGGGGACGCG-3′. Also, a second round of PCR was performed with the first mutagenic PCR product as a template using the forward primer 5′-CTCCCCTGTGAGGAACTA-3′ (JFH1-WT-AgeI-F) and the reverse primer 5′-AGGTAAGCAGGCC CAAGC-3′ (JFH1-WT-KpnI-R) to make a recombinant construct, which was cloned into the JFH1 E2p7_5A/5B_GFP plasmid. The Q257G mutation was also introduced into the E1 region of the TN strain (genotype 1a) by site-directed mutagenesis with the following mutagenic primer set: forward, 5′-CAAACTCCCCACAACGGGGCTTCGACGTCACATC-3′ and reverse, 5′-GATGTGACGTCGAAGCCCCGTTGTGGGGAGT TTG-3′.

### Antiviral assay with drug combinations *in vitro*

The effects of drug combinations on antiviral activity *in vitro* were assessed with minor modifications of dose–response curve (DRC) analysis. The DAAs of interest (telaprevir, daclatasvir, and sofosbuvir) and IFN-α were tested individually or in combination with IDPP. In brief, Huh-7.5 cells were treated with suboptimal (8-fold EC_50,_ 4-fold EC_50,_ 2-fold EC_50,_ EC_50,_ 1/2-fold EC_50,_ 1/4-fold EC_50_, and 1/8-fold EC_50_) concentrations of IDPP and HCV inhibitors (telaprevir, daclatasvir, sofosbuvir and IFN-α), respectively. Antiviral activities of each combination were analyzed at 72 h post treatment. Data were analyzed using CompuSyn software (ComboSyn, Inc.), and the combination index (CI) was determined as described^29^.

### Statistical analysis

Experimental data were normalized, and the statistical significance of differences was determined by Student’s *t*-tests. Differences were considered significant when *p* ≤ 0.05 (indicated by asterisks).

## Additional Information

**How to cite this article**: Lee, M. *et al*. A Novel Inhibitor IDPP Interferes with Entry and Egress of HCV by Targeting Glycoprotein E1 in a Genotype-Specific Manner. *Sci. Rep.* **7**, 44676; doi: 10.1038/srep44676 (2017).

**Publisher's note:** Springer Nature remains neutral with regard to jurisdictional claims in published maps and institutional affiliations.

## Supplementary Material

Supplementary Table 1 and Figures

## Figures and Tables

**Table 1:** Antiviral activity of IDPP to different HCV genotypes.

Virus strain (Core-NS2)^a^	Genotype	E1 (257aa)^b^	EC50 [μM]^c^
JFH1_AB047639	2a	G	0.00006
Jc1 (J6/JFH1)	2a	G	0.004
J8 (J8/JFH1)	2b	S	0.05
TN	1a	Q	>1
S52	3a	S	>1
ED43	4a	S	>1
SA13	5a	P	>1
HK6a	6a	T	>1
QC69	7a	D	>1

^a^Given virus strains refer to PubMed (DDBJ/EMBL/GenBank accession no AB047639).

Various HCV chimeras were utilized to determine EC50 values^28^.

^b^Amino acid at position 257 in E1.

^c^EC50 values determined by dose–response curve analysis using Prism v5.0 software (Graph Pad Software, Inc., La Jolla, CA).

**Table 2:** Synergistic inhibitory effects of IDPP with other HCV inhibitors.

Drug Combination	Combination ratio^a^	CI^b^ at inhibition of				Mean CI ± SD^c^	Description^d^
		50%	75%	90%	95%		
IDPP + Telaprevir	1:0.0001	0.833	0.841	0.866	0.889	0.87 ± 0.01	Synergism
IDPP + Daclatasvir	1:2	0.769	0.581	0.485	0.404	0.50 ± 0.00	Synergism
IDPP + Sofosbuvir	1:0.0002	1.100	1.032	0.991	0.972	1.00 ± 0.04	Additive
IDPP + IFN-α	1:0.00002	0.622	0.365	0.214	0.149	0.26 ± 0.00	Synergism

^a^Combination ratio of two drugs as depicted.

^b^CI values were determined by isobologram equations: CI = [(D)1/(D*x*)1] + [(D)2/(D*x*)2], where D*x* = D*m*[*f*a/(1-*f*a)]^1/*m*^ as described previously^29^.

^c^Mean CI values were determined by following formula: [CI_50_ + 2CI_75_ + 3CI_90_ + 4CI_95_]/10. Data represent the average CI value ± SD from two independent assays (n = 2).

^d^Description of CI values: synergism (CI ≤ 0.9), additive effect (0.9 < CI < 1.1), and antagonism (CI ≥ 1.1).

**Table 3:** Evaluation of IDPP inhibitory effect on DAA-resistant HCV variants.

Inhibitors	Drug-resistant variants (RV)^a^			EC_50_ (nM)^b^		Resistance (fold)^c^
	NS3	NS5A	NS5B	WT	RV	
Telaprevir (VX-950)	A156T	─	─	390 ± 40	6650 ± 4740	17.1
Daclatasvir (BMS-790052)	─	L31M	─	0.00186 ± 0.00004	60.21 ± 1.7	3237.1
Sofosbuvir (PSI-7977)	─	─	T179A/S282T/M289L/I293L	250 ± 40	4350 ± 1480	17.4
IDPP	A156T	─	─	0.086 ± 0.091	0.078 ± 0.032	0.91
IDPP	─	L31M	─		0.819 ± 0.57	9.20
IDPP	─	─	T179A/S282T/M289L/I293L		0.090 ± 0.066	1.04

^a^Each drug resistance HCVcc (JFH1) mutant harbors amino acid change(s) at indicated site(s).

^b^EC_50_ values were determined by DRC analysis as described in our manuscript. Data represent mean ± SD (n ≥ 2).

^c^Resistance fold indicates the ratio of resistant virus (RV) EC_50_ to wild type (WT) EC_50_.
