# Supplementary material for: A Novel Inhibitor IDPP Interferes with Entry and Egress of HCV by Targeting Glycoprotein E1 in a Genotype-Specific Manner
Source: Sci Rep. 2017 Mar 23;7:44676. doi: 10.1038/srep44676 (PMC5363083; doi:10.1038/srep44676)
Supplement: Supplementary Table 1 and Figures [file srep44676-s1.docx]

**Supporting Information manuscript to:**

**A Novel Inhibitor IDPP Interferes with Entry and Egress of HCV by Targeting Glycoprotein E1 in a Genotype-Specific Manner**

Myungeun Lee, Jaewon Yang, Eunji Jo, Ji-Young Lee, Hee-Young Kim, Ralf Bartenschlager, Eui-Cheol Shin, Yong-Soo Bae, and Marc P. Windisch

**Supporting Table**

| **S1 Table.**  **S1 Table. Comparison of infectivity between wild-type and IDPP-resistant viruses generated by site-directed mutagenesis.** | | | | |
| --- | --- | --- | --- | --- |
| **Protein residues^a^** | Genotype | E1 | | **Infectivity relative to wild-type (%)^b,c^** |
|  |  | **250-260aa** | **341-350aa** |  |
| **JFH1_AB047639** | **2a** | **PGALTQGLRT** | **PEVIIDIVSG** | **100.0 ± 00.0** |
| **G257R** | **2a** | **PGALTQRLRT** | **PEVIIDIVSG** | **109.5 ± 10.8** |
| **V343A** | **2a** | **PGALTQGLRT** | **PEAIIDIVSG** | **108.4 ± 8.30** |
| **G257R_V343A** | **2a** | **PGALTQRLRT** | **PEAIIDIVSG** | **089.7 ± 3.40** |
| ^a^The given amino acid positions refer to the JFH1 consensus genome (DDBJ/EMBL/GenBank accession no. AB047639). | | | | |
| ^b^Values represent the mean ± S.D. of two independent experiments. | | | | |
| ^c^Infectivity is presented as the percent (%) of infection level of IDPP-resistant to wild type virus. | | | | |

**Supporting Figures**

**S1 Fig.**

IDPP [IminoDiPyridinoPyrimidine]

**S1 Fig. Molecular structure of IDPP (IminoDiPyridinoPyrimidine).**

**S2 Fig.**

**
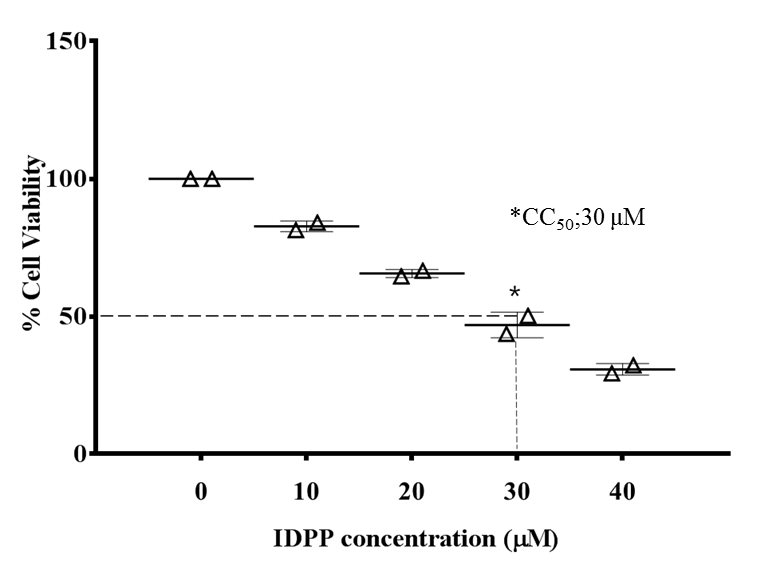
**

**S2 Fig. Cytotoxicity of IDPP.** The 50% cytotoxic concentration (CC_50_) of IDPP was determined by cell viability in cell cultures in the presence of IDPP at concentrations up to 40 μM. The CC_50_ value was calculated using Prism v 5.0c software (Graph Pad Software, Inc., La Jolla, CA). Dotted line indicates the CC_50_ value (CC_50_= 30 μM).

**S3 Fig.**


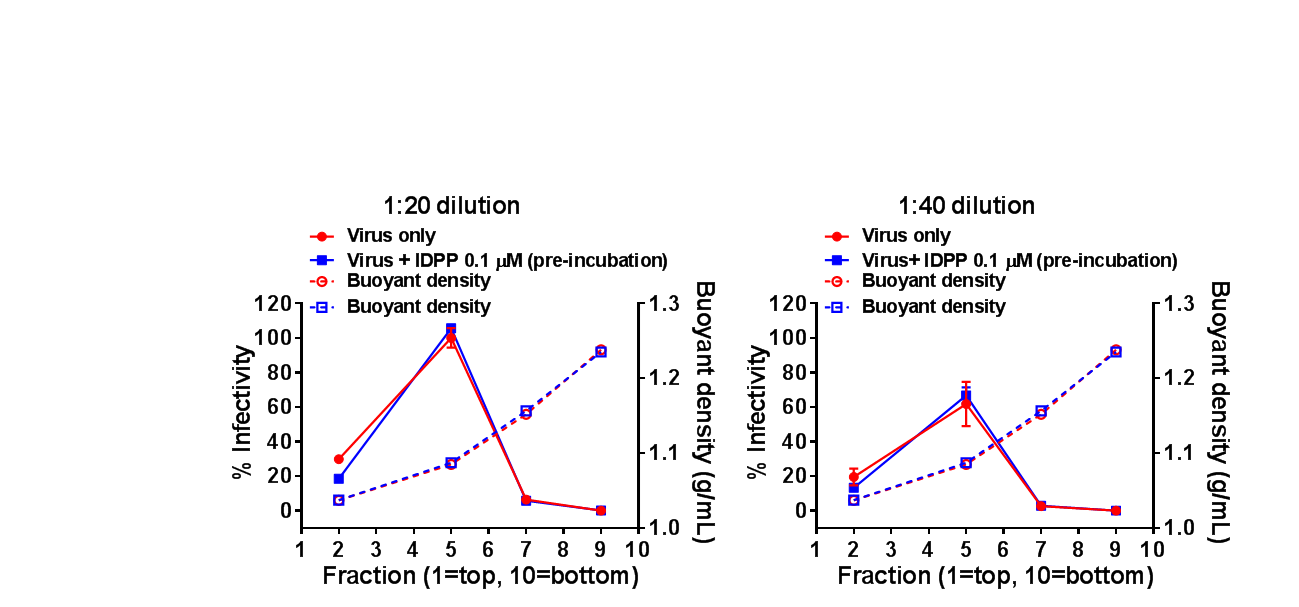


**S3 Fig. Carry-over effects of residual IDPP after ultracentrifugation.** HCVcc (JFH1; NS5A-GFP) was pre-incubated with or without 0.1 μM IDPP at RT for 1 h, and then loaded on and fractionated in 20% to 80% sucrose density gradient ultracentrifugation. All fractions were diluted at 1:20 and 1:40 in PBS, and the infectivity of each fraction was determined by measuring GFP-positive cells in the 2^nd^ infection. Data represent the relative infectivity of each fraction to that of the untreated control fraction at the same buoyant density. Pre-incubation of virus with IDPP or different dilution rates did not make any changes in the infectivity of the original virus once fractionated by density gradient ultracentrifugation, suggesting that there is no room for carry-over effects of the residual IDPP on the 2^nd^ infection once samples are fractionated by a density gradient ultracentrifugation.

**S4 Fig.**

**B**

**A**

**Huh-7 transiently transfected with viral genome (JFH1)**

**HCV core**

**ApoE (Intra)**


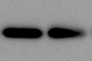

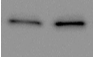


**1.0**

**1.0**

**0.6**

**1.3**

**α-tubulin**


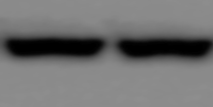


**IDPP (1 μM)**

**-**

**+**

**Huh-7 infected with HCVcc**

**(JFH1)**

**HCVcc**

**-**

**+**

**+**

**+**

**+**

**+**

**+**

**+**

**+**

**DMSO**

**IDPP (nM)**

**1000**

**100**

**10**

**1**

**0.1**

**0.01**

**-**

**-**


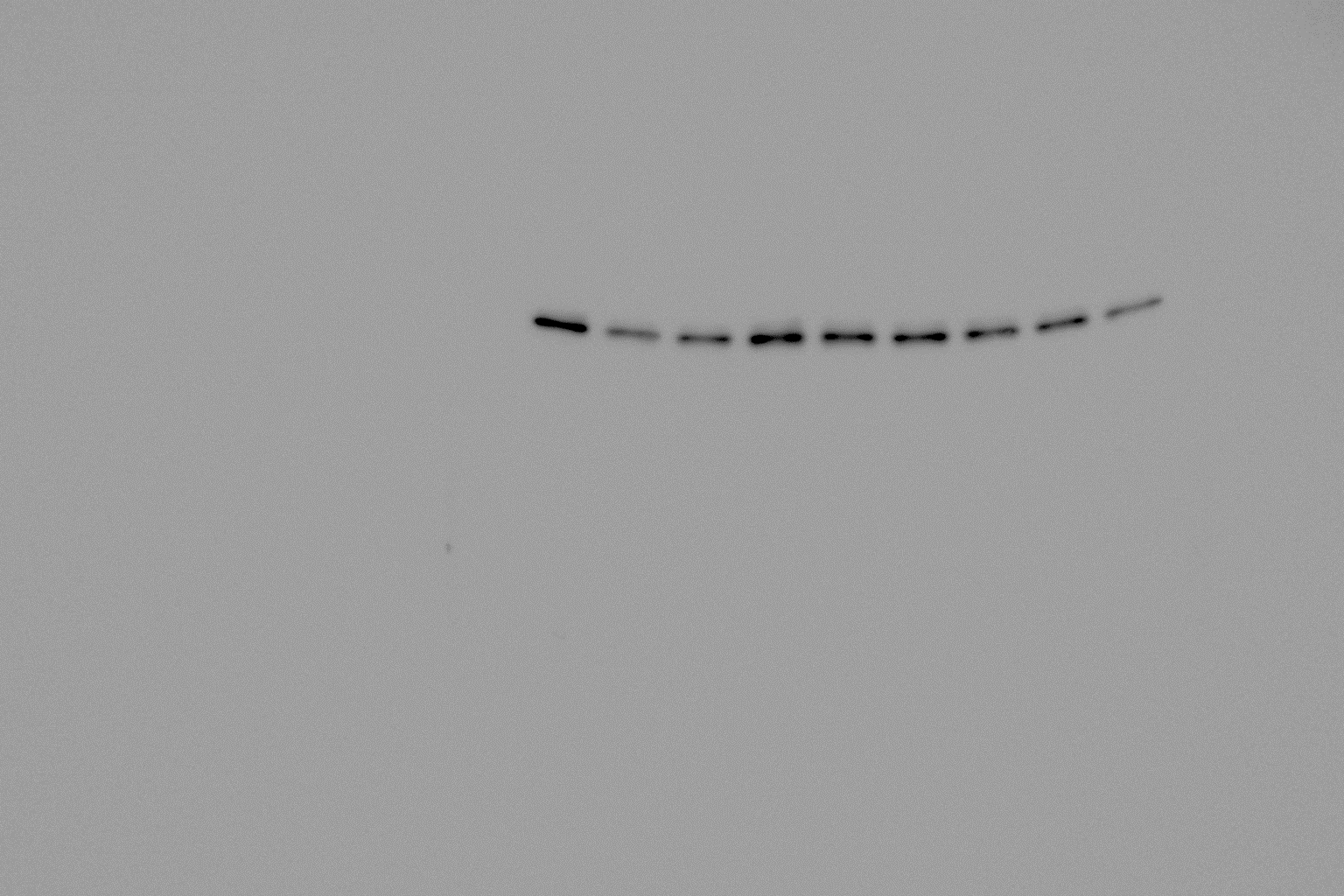


**ApoE**

**(Intra)**


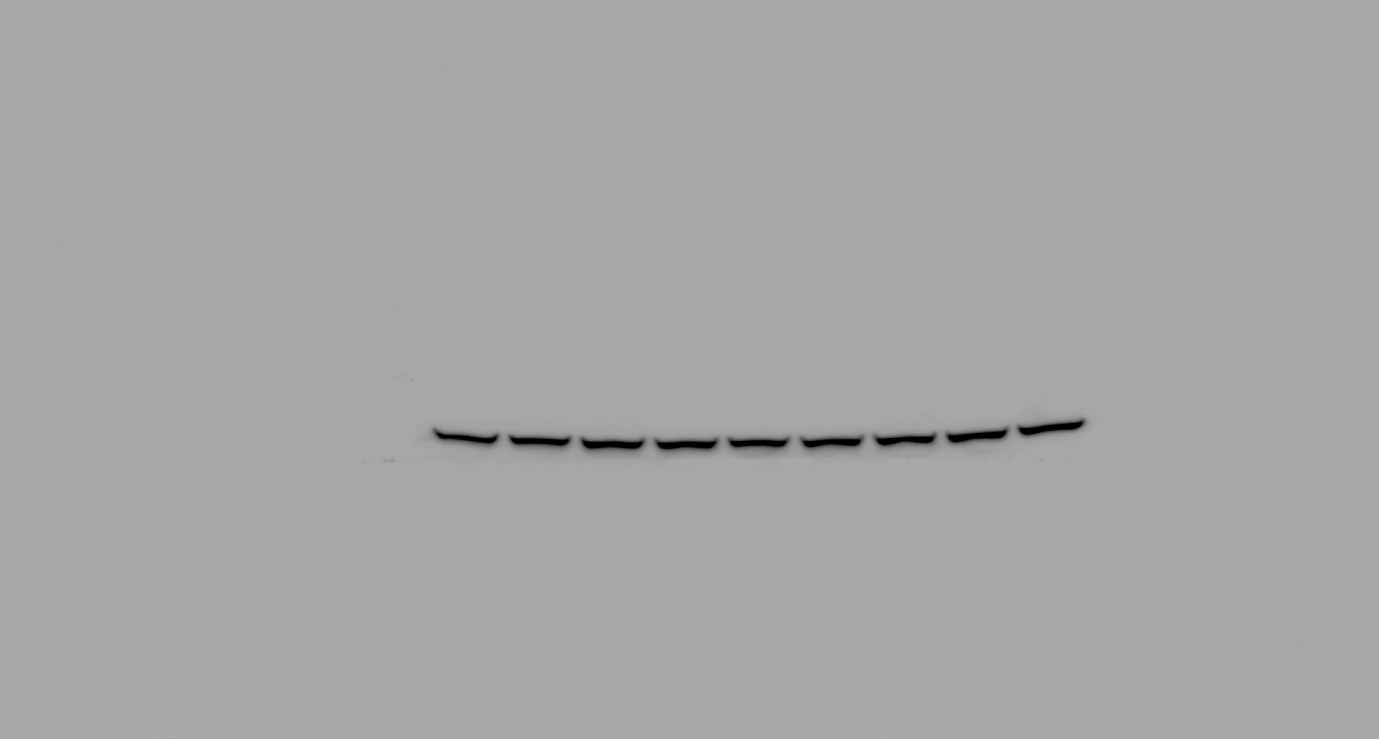


**ɑ-tubulin**

**1.0**

**0.37**

**0.45**

**0.93**

**0.77**

**0.74**

**0.55**

**0.48**

**0.26**

**S4 Fig. Intracellular ApoE levels after IDPP treatment.** Huh-7.5 cells infected with HCVcc (**A**) or transiently transfected HCV genomic RNAs (**B**) were treated with different concentrations of IDPP for 72 h. Intracellular ApoE levels were assessed by Western blot (α-ApoE).

**S5 Fig.**

**
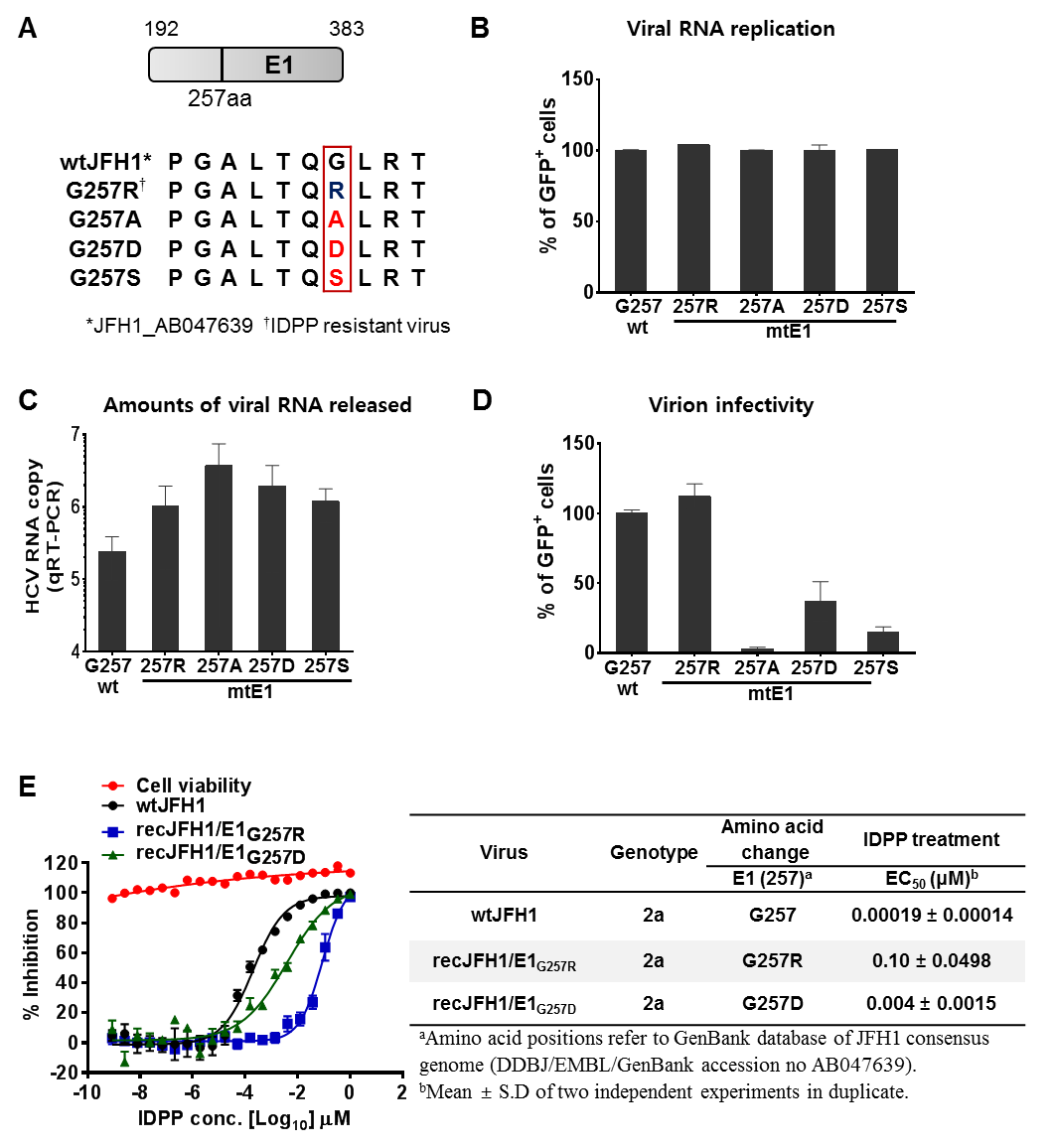
**

**S5 Fig. Characterization of recombinant JFH1 viruses with a mutation at Gly-257 residue in the E1 glycoprotein.** (**A**) Amino acid sequence surrounding the 257 residue of E1 glycoproteins in wild type (wt) JFH1 and IDPP resistant mutant (mtE1) viruses. Box indicates the amino acid residues of each mutant at site 257. (**B**) Huh-7.5 cells transfected with GFP-integrated wt and mtE1 JFH1 viral genome. GFP-positive cells, indicating viral replication after transfection, were counted, and are represented as a percentage of the wt control. (**C**) The numbers of HCV RNA copies in the supernatants of the transfected cultures were assessed by qRT-PCR 3 days after transfection. (**D**) The wt and mtE1 viruses harvested from the cultures were transferred onto fresh Huh-7.5 cells, and GFP^+^ cells were counted at 72 h p.i. (**E**) IDPP sensitivity of wt and mtE1 strains as represented by DRC. The EC_50_ of each strain was determined by Hill equation analysis using Prism v 5.0c software.

**S6 Fig.**

**
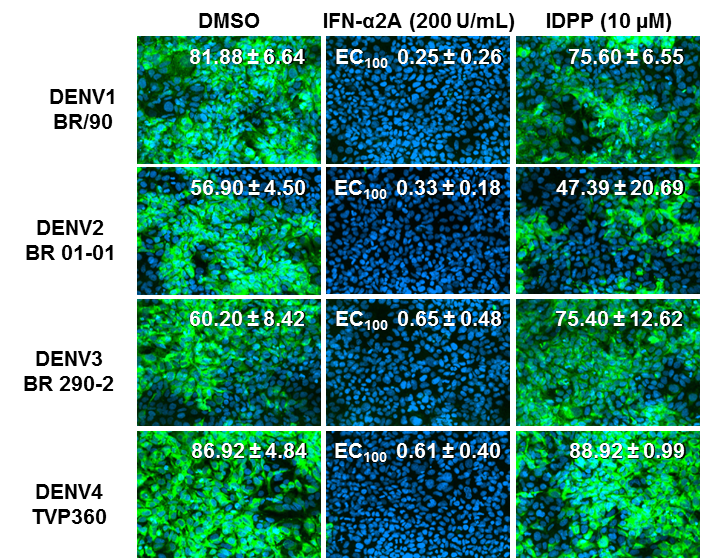
**

**S6 Fig. Effects of IDPP on Dengue virus (DENV) infection.** The antiviral activity of IDPP on Dengue virus was examined. Huh-7.5 cells were pretreated with 10 µM IDPP, IFN-α2A, or DMSO (control), and then inoculated with DENV1, DENV2, DENV3, or DENV4 at an M.O.I. of 0.5, and cultured for 96 h. The number of DENV-infected cells was assessed by measuring the expression of DENV E protein after immunostaining with Alexa-488 (green). Data represent mean ± SD of the percentage of GFP-positive cells.

**S7 Fig.**

**
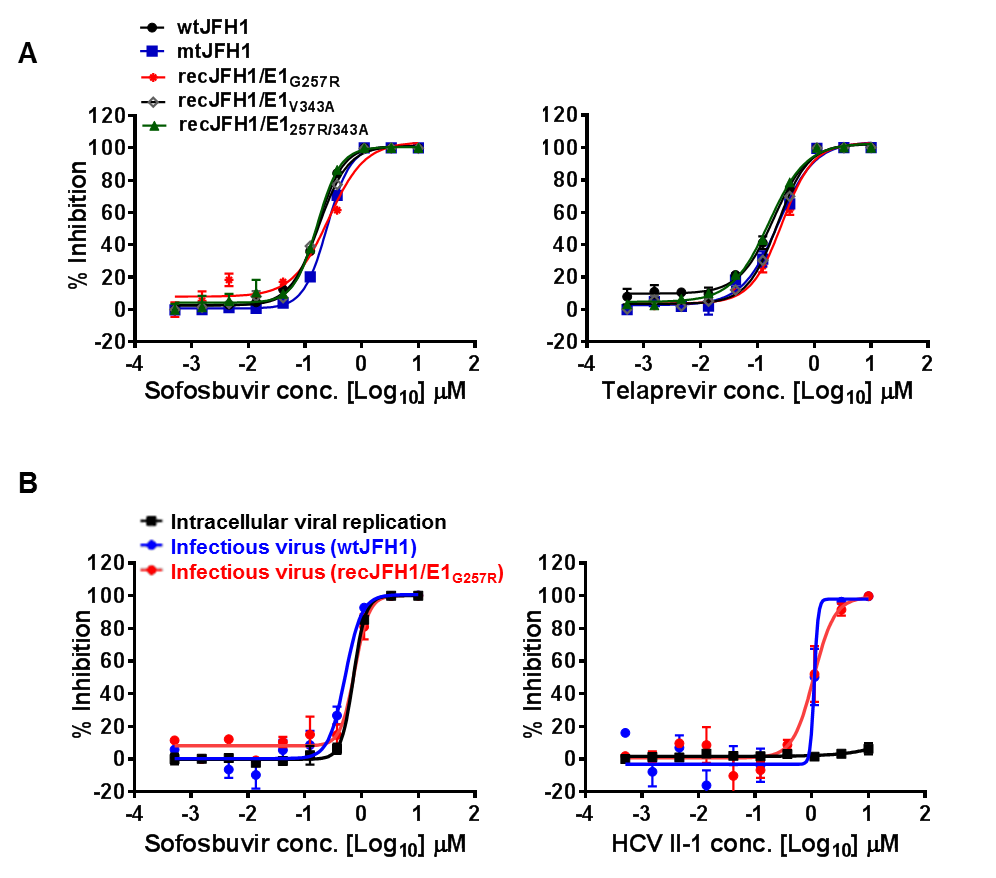
**

**S7 Fig. Evaluation of antiviral activity of HCV drugs with IDPP-resistant mutant.** (**A**) Huh-7.5 cells infected with IDPP-resistant mutant (mtJFH1) and recombinant viruses (with amino acid substitutions at residues 257 and/or 343 generated *via* site-directed mutagenesis) were cultured in the presence of HCV inhibitors (sofosbuvir and telaprevir). Percent of inhibition is shown by the DRC plotted with the mean ± SD of two independent experiments done in duplicate. (**B**) Huh-7.5 cells were transiently transfected with wild type (wtJFH1) or mutant (recJFH1/E1_G257R_) viral RNA and cultured for 2 days in the presence of HCV-inhibiting drugs (sofosbuvir and entry inhibitor HCV II-1 [^1^](#_ENREF_1)). Intracellular viral RNA and extracellular viral particles in the culture supernatants were quantified by measuring the number of GFP positive cells.

**References**

1 Bush, C. O. *et al.* A small-molecule inhibitor of hepatitis C virus infectivity. *Antimicrobial agents and chemotherapy* **58**, 386-396, doi:10.1128/aac.02083-13 (2014).
